# Refined Division of Sleep Stages in the Mouse Based on Distributed Deep Electrodes and Underlying Infra‐Slow Oscillation

**DOI:** 10.1111/jsr.70262

**Published:** 2025-12-15

**Authors:** Nanxiang Jin, Sara Häkli, Keerthana Malathi, Hennariikka Koivisto, Theodor Boc, Jonas Schimmel, Eric Castell‐Caubet, Irina Gureviciene, Heikki Tanila

**Affiliations:** ^1^ A. I. Virtanen Institute University of Eastern Finland Kuopio Finland

**Keywords:** delta‐wave, EEG, noradrenaline, norepinephrine, REM, sleep‐spindle, theta

## Abstract

The mouse sleep is mostly recorded with only epidural electrodes and divided simply into NREM and REM stages. With the help of distributed intracerebral triplet electrodes, we searched for possible new electrophysiological signatures to characterise more specific sleep substages within the timeframe of seconds to tens of minutes. We implanted 17 C57BL/6J male mice with double or triple wire electrodes into the hippocampus, somatosensory cortex and olfactory bulb, conventional skull screw electrodes and neck EMG electrodes. Sessions with at least 1 h of sleep of the 3‐h total recording time were included in the analysis. We could identify N1, N2 and N3 stages in the mouse NREM. N2 could be further divided into N2s with spindles and N2n without spindles. Furthermore, N3 following N2n vs. N2s showed different features and were therefore coined N3s and N3n, respectively. The REM could be divided into asynchronous (aREM), synchronous (sREM) and phasic (pREM) substages. The EEG power between 5 and 30 Hz showed quasi‐periodic fluctuation at an infra‐slow range (0.005–0.02 Hz). The sequence of sleep stages followed reliably this fluctuation, so that the onset of N2n co‐occurred with a low power, N2s halfway on the rise, aREM or N3s near the peak and sREM on the descending phase. The infra‐slow fluctuation was temporarily abolished by damaging the locus coeruleus noradrenergic axons with the selective neurotoxin DSP‐4 or by administration of the alpha‐2 adrenergic agonist medetomidine. As a result, the brain got locked in a continuous N3‐like state and no REM was present.

## 1 Introduction

The established method to divide rodent sleep into stages is to record epidural EEG and neck EMG and to analyse the data in 10 s sweeps, which corresponds to the established protocol to rate the human EEG in 30‐s epochs and is also a convenient frame for Fourier transformation. It is customary to coin periods with high EMG and low EEG amplitude as wake and the rest as sleep. Periods of very low EMG and high theta/delta ratio are considered REM sleep and the opposite pattern NREM sleep (Soltani et al. 2019; Kent et al. 2019). The similar EEG/EMG and EEG frequency band ratios have been used as the basis of various automated sleep scoring systems for rats and mice (Benington et al. 1994; Louis et al. 2004; Stephenson et al. 2009; Rytkönen et al. 2011). These algorithms work well to separate the main sleep stages NREM and REM, and to some extent make a distinction between light and deep NREM sleep (slow‐wave sleep). However, there is prevailing uncertainty about the criteria for NREM substages, especially about the wake—N1 transition (Bagur et al. 2018) and the N3 stage (Lacroix et al. 2018). Further, the onset of REM sleep is not sharp and often a transition period is identified (Gottesmann 1996).

If one is interested in translational studies to understand the neural underpinnings of human sleep regulation by using genetically modified mouse models, there are obvious gaps in our knowledge. First, while mouse sleep scoring is based on dividing the EEG into certain frequency bands and their ratios, clinical human EEG is still based on identifying key graphoelements and assessing their frequency of occurrence in the traditional 30‐s fixed timeframe. From the translational viewpoint, it would be helpful to also identify specific graphoelements for each sleep stage in the mouse. Despite extensive literature searches, we have not come across a paper that describes such graphoelements in the mouse EEG, except cortical spindles and hippocampal theta. A one‐to‐one correspondence of these graphoelements is probably impossible due to species differences. For instance, the attenuation of occipital alpha rhythm is a key criterion for the wave—N1 sleep transition (Berry et al. 2015), but occipital alpha does not exist in mice. Nevertheless, the mouse EEG may have a corresponding graphoelement that would serve the same purpose. In fact, a recent study demonstrated the utility of mouse olfactory bulb (OB) recordings in differentiating between wake and light sleep, an approach never used in human sleep recordings (Bagur et al. 2018). Further, REM sleep in humans is mainly defined by the robust changes in the electro‐oculogram, which is seldom used in mouse recording. On the other hand, the possibility to directly record hippocampal theta with intracranial electrodes provides a far more accurate means of assessing the onset and possible variations of REM sleep than the scalp recording combined with EOG in humans.

Recent advances in optogenetics in genetically modified mice have made it possible to turn on and off an individual group of specified neurons at a millisecond time resolution and assess their contribution to sleep regulation. Thereby, the estimated number of brain nuclei involved in sleep–wake regulation has constantly increased and approaches as many as 30 (Liu and Dan 2019). To relate changes in neuronal activity in any of these nuclei to certain sleep phases is currently mainly limited by two factors. First, the common sleep staging into NREM and REM sleep is far too simple as a reference. Second, the typical approach based on FFT on a 10 s timeframe may be too coarse to detect transitions that last only for a few seconds.

In this study, we aimed to improve the identification of different sleep stages in the mouse by searching for more stage‐specific graphoelements using multisite intracranial recording of local field potentials. We recorded skull EEG, intracortical, OB and hippocampal local field potentials and EMG of neck muscles in freely moving mice during their sleep and looked for transitions in characteristic electrophysiological signatures. In addition, we monitored EEG both at time scales of seconds and tens of minutes and found that the depth of sleep correlates with an infra‐slow quasi‐periodic power fluctuation of spindle and delta frequency bands at all recording sites. Since recent data have linked the spindle‐band power variation with the activity of locus coeruleus (LC) neurons and fluctuations of cortical noradrenaline (NA) levels (Osorio‐Forero et al. 2021; Kjaerby et al. 2022), we tested if neurotoxic lesioning or pharmacological blockade of LC activity could dampen the brain‐wide slow oscillation. This proved to be the case.

## 2 Material and Methods

### 2.1 Animals

Sixteen C57BL/6J male mice bred at the Laboratory Animal Facilities of University of Eastern Finland were used in the study, 8–12 for sleep staging and five for pharmacological interventions. The mice were between 4 and 8 months during the recordings. The mice were housed in a controlled environment (temperature 22°C ± 1°C, humidity 50%–60%, lights on 07:00–19:00) with food and water available ad libitum. All animal procedures were carried out in accordance with the EU Directive 2010/63 and approved by the Animal Experiment Board of Finland.

### 2.2 Electrode Implantation

The mice were implanted with double or triple wire electrodes into the hippocampus, somatosensory cortex and OB and with conventional skull screw electrodes above the frontal cortex. Screw electrodes above the cerebellum worked as the ground and reference, and also as anchors for dental acrylic cement for a miniature connector. Electromyogram (EMG) was recorded with a 150 μm stainless‐steel wire between the neck muscles. Under isoflurane anaesthesia (4.5%–2%) two cortical screw electrodes were attached to the left and right frontal bone (AP +1.7, ML ±1.8 from bregma). A bundle of three 50 μm insulated stainless‐steel wire electrodes (stagger 400 μm between the tips) was inserted into the left somatosensory cortex (AP −0.5, ML +1.5, DV −1.0 from dura), a triple electrode with 220 μm stagger into the left hippocampus (AP −2.2, ML +1.5 from bregma, DV −1.3 from dura) and another triplet electrode with 400 μm stagger into the right hippocampus (AP −2.2, ML −1.5 from bregma, DV −1.7 from dura). Furthermore, a doublet wire electrode (stagger 500 μm) was inserted into the left OB (AP +4.2, ML +1.0 from bregma, DV −0.6 from dura mater surface). The location of the deep electrodes was confirmed by electrophysiological landmarks and histologically (Figure S1A,B). Carprofen (5 mg/kg, i.p.) was given for postoperative analgesia. The mice were given 1 week of recovery after the operation before they were familiarised with the recording environment.

### 2.3 Video‐EEG Acquisition

After a 1 week‐period of recovery from the surgery, the mice were first familiarised with a copy the recording environment for three 1‐h sessions and then had two full recording sessions to ensure a normal sleep in the video‐EEG room before starting the actual experiment. The video‐EEG recording took place in a circular frame made of brown hardboard (diameter 18.5 cm, wall height 18 cm) on a translucent glass plate that was illuminated from below. Each mouse was recorded individually. A light recording cable with a counterweight through a pulley system was attached fixed to a preamplifier (Plexon) that connected to the imbedded connector on the mouse's head. The other end of the recording cable was connected to an AC amplifier (A‐M Systems; gain 1000, analogue band‐pass 1–3000 Hz). The amplified signal was digitised at 2 kHz per channel. The movements of the animals were recorded with an overhead video camera. Synchronised EEG and video signals were acquired with SciWorks 5.0 programme (A‐M Systems). Since the mice tended to have a preferred rotation direction the multichannel recording cable got twisted, which limited the recording time. Each recording session took 3 h, either in the morning or afternoon, but always during the light period.

### 2.4 Pharmacological Manipulations

The LC selective toxin of noradrenergic axons (Grzanna et al. 1989), DSP‐4 (N‐(2‐chloroethyl)‐N‐ethyl‐2‐bromobenzylamine), was dissolved in 0.9% saline and injected intraperitoneally at a dose of 50 mg/kg. First, the drug was injected twice, with 3 days between the injections. In three mice, a third injection followed after 3 weeks, in the remaining three mice after 10 days. Finally, a fourth injection was given 3 weeks after the third injection to all mice. After the second injection, the mice were recorded 1 and 3 days after each injection.

The selective α2‐adrenergic agonist, medetomidine, was injected intraperitoneally at the doses of 40 and 60 μg just before the onset of a 3‐h sleep–EEG recording.

### 2.5 Histology

At the end of the experiment, positive DC current was passed through the wire electrodes under deep pentobarbital‐chloralhydrate anaesthesia (pentobarbital 50 mg/kg + chloralhydrate 200 mg/kg i.p.). The mouse was perfused through the heart first with ice‐cold saline to rinse blood from the cerebral circulation and then with 4% paraformaldehyde solution. Then the brain was removed and immersion‐fixed for 4 h in 4% paraformaldehyde solution, followed by 30% sucrose overnight. The brain was left in antifreeze at −20°C until cut into 35 μm coronal sections with a freezing slide microtome. The electrode tips were identified by the Prussian blue reaction for the released iron in the tissue.

The DSP‐4 effect was verified by tyrosine hydroxylase (TH) staining of the parietal cortex that does not have a significant amount of dopamine fibres. Two sections at the level of the visual cortex were stained for TH. Sections were rinsed with Tris‐Buffered Saline with Triton (TBS‐T) and pretreated with 10% normal goat serum (NGS, Biowest, Nuaillé, France, cat.no S2000) in TBS‐T for 30 min. Sections were stained with polyclonal rabbit anti‐TH antibody (anti‐TH, 1:7000, Merck, Temecula, CA, USA, cat.no AB152) in 1% NGS and incubated for 72 h at 4°C on a shaker table. Following this, sections were rinsed and incubated for 2 h with the secondary antibody, biotinylated goat antirabbit (1:500, Vector Laboratories, Burlingame, CA, USA, cat.no BA‐1000). After this, sections were rinsed and transferred to Streptavidin‐horseradish peroxidase conjugate (1:1000, GE Healthcare, Buckinghamshire, UK, cat.no RPN1231V) solution for 2 h. Visualisation of noradrenergic neurons (and few dopaminergic) was achieved by incubation with DAB–Ni solution. Stained sections were rinsed with PBS and mounted on gelatin‐coated slides, cleared with xylene (VWR International, Helsinki, Finland) and mounted in DePeX (BDH Chemicals via VWR International, Helsinki, Finland).

### 2.6 Data Analysis

Power spectral density (PSD) of each EEG/LFP channel was calculated from 1 to 100 Hz with pWelch method (default setting) in Matlab based on 10‐s epochs. To help visualisation of the data, we built a custom interactive Matlab‐based tool (‘PSD Scope’) that plotted the PSD of EEG/LFP in four select channels as a heatmap and the EMG amplitude during the entire 3‐h recording. First, a rough estimate was made on the sleep time based on the PSD over the entire 3‐h recording in the cortical screw channel and the EMG. Only sessions with > 1 h of sleep were included in the main analysis.

Second, experienced mouse‐EEG readers (N.J., H.T.) screened the recordings using a multichannel EEG/LFP/EMG display (a custom‐made Matlab‐based software) and the PSD Scope and annotated all state transitions. With state transition, we mean a clear change in EEG/LFP signatures that was visible in more than one recording site, or in the case of cortex and hippocampus with three electrodes, in all channels in either location. In addition, the new stage had to last at least 3 s. Thereafter, we assigned the epochs between the transitions to established vigilance stages: movement, waking immobility, NREM stages N1, N2 and N3, and REM, and further to substages based on stage‐specific signatures described in the literature. These are explained more in detail in the Results. Next, one representative 3‐h sleep session of eight mice was carefully annotated according to the identified sleep stages and substages so that each stage transition, its type and time (in seconds) was separately marked in a spreadsheet. The transitions were first counted for each mouse and then summed across all mice.

To verify if every newly defined sleep stage and substage was indeed statistically distinguishable from others, we compared the PSD in select frequency bands around observed PSD peaks between the sleep stages and substages. First, all EEG/LFP epochs of the same (sub)stage were extracted according to the annotations and connected into a single continuum. Second, each connected EEG/LFP was cut into 10‐s epochs, and then the PSD of each epoch was calculated from 1 to 100 Hz with the pWelch method (default setting) and averaged by epoch. To minimise the influence of 50 Hz line noise on the power analysis, the PSD at 50 Hz was replaced by the mean of 49 and 51 Hz. Third, the PSD values of each selected frequency band were summed up within the same channel and the same (sub)stage and thereafter fed into statistical tests for within‐subject pairwise comparisons between (sub)stages. For visualisation of figures, we first normalised the PSD of each substage in a given mouse to its total PSD, but left it undone for the statistical testing which had a within‐subject design and thus was not influenced by normalisation.

The statistical analysis was conducted according to the following steps. First, Levene's test was used to test the homogeneity of variances and Shapiro–Wilk test to test the normality of distribution in each group. If the data followed a normal distribution and exhibited homogeneity of variance, we tested differences between sleep (sub)stages with ANOVA for repeated measures (ANOVA‐RM). The threshold for significance was set at *p* < 0.05. If Mauchly's tests for sphericity yielded *p* < 0.05, the results needed corrections for departure from sphericity (Greenhouse–Geisser or Huynh‐Feldt correction). Finally, a post hoc comparison was done with Student's t‐test between every pair and *p* values were adjusted for multiple comparisons by the Sidak method. However, if the data either failed to follow a normal distribution or showed no homogeneity of variance, we used the nonparametric Friedman test to search for differences among group medians. If the Friedman proved significant (*p* < 0.05), we ran the Nemenyi‐Wilcoxon‐Wilcox all‐pairs test post hoc to find which two groups were different (*p* value adjustment method: single‐step). Levene's and Friedman tests were run in Matlab while ANOVA‐RM and Nemenyi tests were run in R.

**FIGURE 1:**
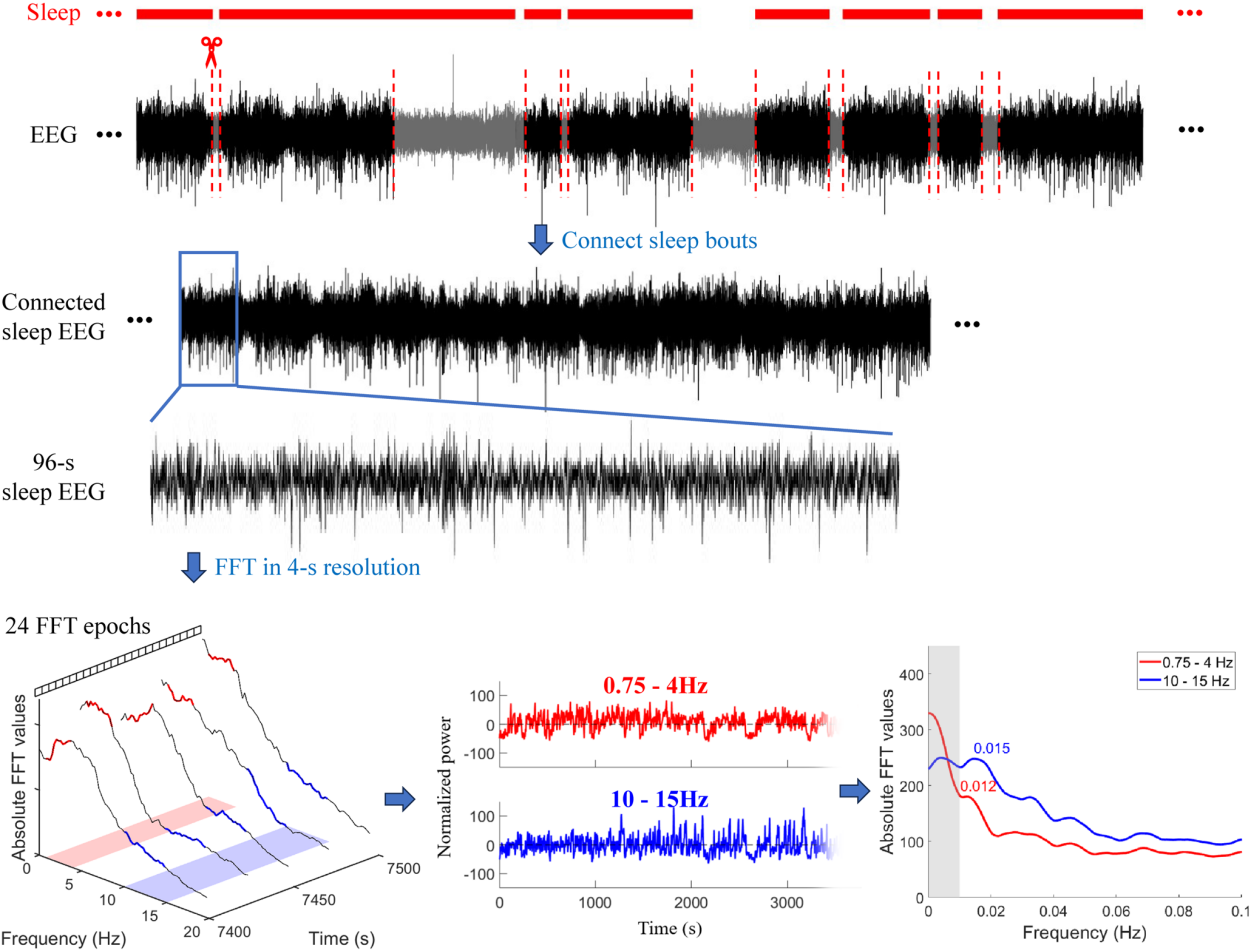
Calculation of the ISO frequency. Based on the EMG amplitude, sleep epochs with low EMG were separated from wake epochs and connected. Then the merged EEG was cut into equal 96‐s epochs and further analysed with fast‐Fourier transformation (FFT) in 4‐s resolution, which returned 24 FFT epochs. Next, the absolute FFT values in 0.75–4 and 10–15 Hz frequency bands in each of the 24 FFT epochs were summed, and the resulting curve was subjected to a second FFT. The power values from the 4‐s FFTs for each frequency band were further normalised to the average NREM sleep FFT of the recording session and plotted against time. The output frequency resolution of FFT was set to 0.001 Hz. The grand average of absolute FFT values from all sleep epochs of all EEG recordings of all mice is shown for 0.75–4 and 10–15 Hz bands.

Examination of the PSD plots for all EEG/LFP channels revealed slow power fluctuations that resembled phase‐amplitude coupling and the previously described infra‐slow brain oscillation (ISO) (Penttonen et al. 1999; Vanhatalo et al. 2004) (see Results *Mouse NREM sleep is modulated by an infra‐slow oscillation*). To search for the frequency of these fluctuations, sleep sessions of 11 mice were detected according to EMG only (to avoid circular reasoning) as follows. First, we band‐pass filtered EMG from 1 to 100 Hz in every recording session and calculated its envelope with Hilbert transform in Matlab. As the EMG signal amplitude and noise level varied between individual mice, we manually adjusted the envelope threshold so that it clearly separated movement activity from background (sleep epochs). In 38 files from 11 mice, the thresholds varied between 0.8 and 2.0 standard deviations above the mean. Third, we excluded the short sleep fragments (< 30 s) and connected neighbouring sleep epochs if the gap in between was < 5 s. Last, we confirmed the detected sleep epochs in video recordings and by the EEG features. To estimate the frequency of the fluctuation, we focused on delta (0.75–4 Hz) and sigma/spindle (10–15 Hz) bands and applied the method described earlier (Lecci et al. 2017). In brief, we focused on the frontal cortical channel (screw electrode) and connected all sleep epochs (including the REM) within each recording session and then cut the entire sleep into 96‐s epochs. Each sleep epoch was fast‐Fourier‐transformed (FFT) in 4‐s resolution, which returned 24 FFT epochs/sleep epoch. Next, we summed the absolute FFT values in 0.75–4 Hz and 10–15 Hz in each of the 24 FFT epochs. The power values from the 4‐s FFTs for each frequency band were further normalised to the average NREM sleep FFT of the recording session and plotted against time. The output frequency resolution of FFT was set to 0.001 Hz. The grand average of absolute FFT values from all sleep epochs of all EEG recordings of all mice showed the ISO frequency for 0.75–4 Hz and 10–15 Hz bands, respectively (Figure 1).

Based on the ISO results, we further asked if the 10–15 Hz ISO was stable during the 3‐h sleep recording or developed along the accumulated sleep. To this end, we selected EEG recordings that contained at least 1 h of sleep and compared the 10–15 Hz ISO between the first (early phase) and last (late phase) 20 min of sleep. The ISO FFT curves were calculated as described above but separately for early and late sleep, first in each recording, and then averaged in each mouse and finally averaged over all mice. All peaks in ISO curves were detected with the Matlab function named ‘findpeaks’ with default parameters. The paired t‐test was applied from 0.01 to 0.1 Hz with steps of 0.01 and a post hoc Bonferroni correction was applied to control the error from multiple comparisons. The significance level was set at 0.05.

Finally, we analysed whether the onset of identified sleep (sub)stages (the transitions) falls on a special phase of the ISO. We band‐pass filtered the FFT values of delta (0.75–4 Hz) and sigma (10–15 Hz) bands of all channels with a zero‐phase Butterworth band‐pass filter (order = 4). Based on the observed ISO in our dataset, we focused this analysis on ISO between 0.005–0.015 Hz. Combining Hilbert transform and Matlab function ‘angle’, we calculated the phase of ISO (delta and sigma separately) at every stage transition timestamp. Next, we applied the MATLAB Circular Statistics Toolbox (https://github.com/circstat/circstat‐matlab) to calculate the mean phase vector per mouse per substage, composed of the mean starting phase of all epochs (function ‘circ_mean’) and the concentration of phases/vector length (function ‘circ_r’). As the last step, phase vectors of all eight mice were averaged within each sleep substage.

## 3 Results

**FIGURE 2:**
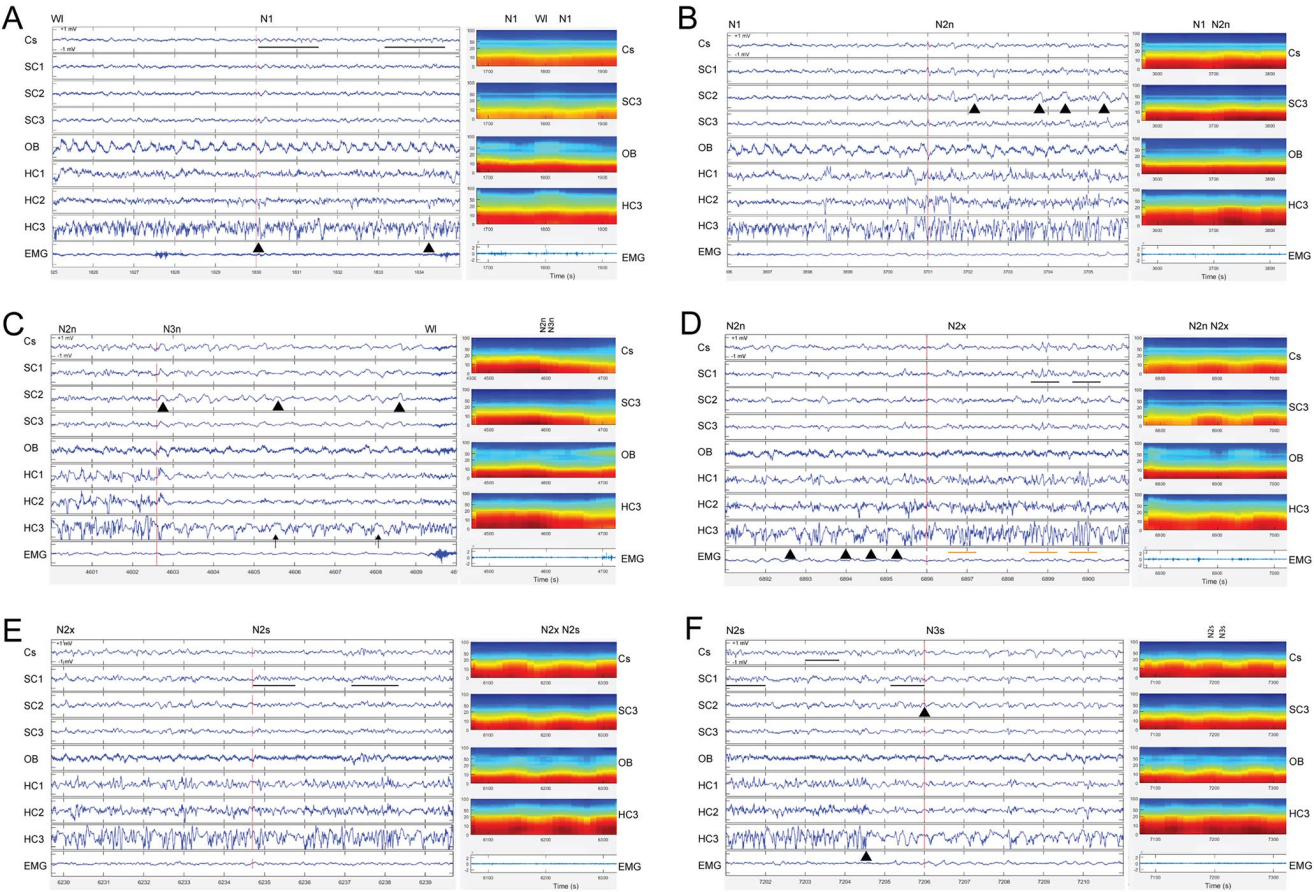
NREM sleep transitions. (A) Waking immobility (WI) to N1 transition is noted in the EEG by the appearance of periodic 6–8 Hz oscillations in the cortical channels (black line) and stable large irregular activity and sharp waves (black triangles) in the hippocampal channels. One can also see the regularity of olfactory bulb (OB) signal reflecting breathing at ~3 Hz during N1. WI is characterised by a prominent 45–65 Hz gamma oscillation in the OB, which disappears during N1. This is difficult to see directly from the raw EEG but is clear in the power spectral density display to the right. (B) N1 to N2n transition is characterised by the appearance of delta‐waves which are best seen in the intracortical channel below cortical layer 4 (black triangles). (C) N2n to N3 transition can be noted by trains of delta‐waves in the intracortical channel (triangles). At the same time, hippocampal faster oscillations are strongly suppressed. The hippocampal delta‐waves are synchronised with the cortical ones to some extent (arrows). (D) N2n to N2x transition involves change in the cortical and especially hippocampal background activity. Hippocampal channels display bursts of large‐amplitude irregular activity at the spindle frequency which co‐occurs with similar irregular activity in the cortical channels (black lines) without clear spindles. (E) N2x to N2s transition. The hippocampal activity remains unchanged but now clear spindles appear locally in the cortex (black lines). (F) N2s to N3s transition. Cortical spindles (black lines) disappear and are replaced by a train of delta activity (black triangle on SC2 row). Intriguingly, the hippocampal irregular spindle frequency activity disappears 1.5 s before the transition in the cortex (black triangle on the HC3 row). Cs, screw electrode on the frontal bone; EMG, electromyogram; HC1‐3, triple wire electrode in the hippocampus; OB, olfactory bulb; SC1‐3, triple wire electrode in the S1 cortex.

**FIGURE 3:**
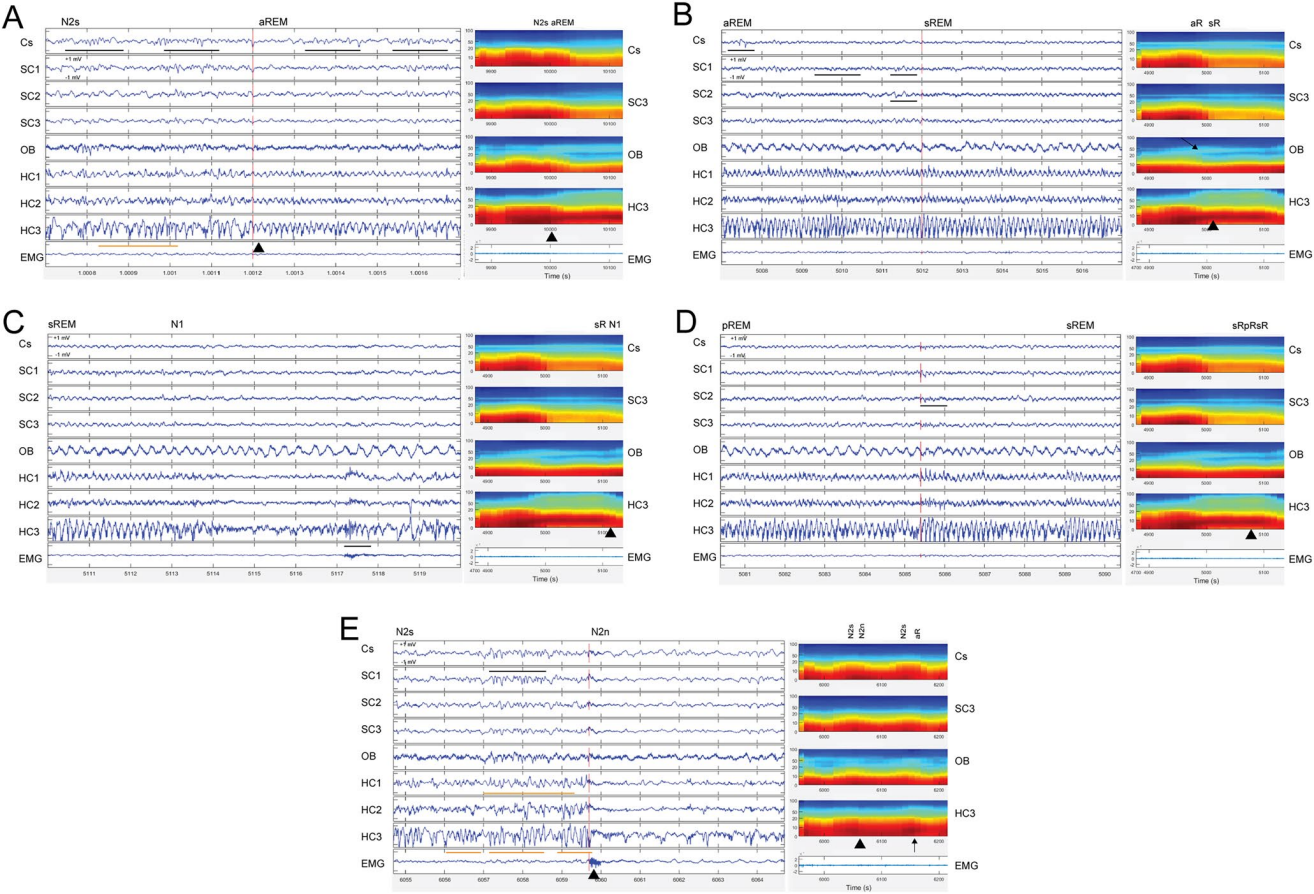
(A) N2s to aREM transition. Theta oscillation appears in the hippocampus, first temporarily (orange line), then more stably (black triangle). In contrast, the cortical channels continue sleep spindle‐like activity (black lines) without any synchronisation with HC theta. The PSD display to the right shows the beginning of strong hippocampal theta oscillation (black triangle). (B) Asynchronous REM (aREM) to synchronous (sREM) transition. While the hippocampus shows continuous and synchronous theta, the cortex continues with spindle‐like and delta‐wave activity (black lines) until the transition point when all channels but the OB get entrained with the hippocampal theta. The OB never displays theta oscillations but maintains its own ~3.5 Hz rhythm reflecting breathing. In the PSD plot to the right, the aREM to sREM transition is also seen as a shift of OB and HC gamma oscillations to a higher frequency (arrow). (C) The end of a sREM episode typically happens all the sudden and proceeds to N1 sleep. Often there is also a microarousal recognised by increased EMG activity (black line). (D) Phasic REM (pREM) is recognised by short bouts of vaxing and waning theta frequency and amplitude in the middle of a sREM epoch. Sometimes burst of high‐frequency activity can be seen intracortically during the fast theta phase (black line on row SC2). (E) Example of a failed aREM start. The theta activity starts in the HC3 but remains irregular and fails to synchronise all HC channels (orange lines). Finally, it ends with a microarousal (triangle) and transition to N2n sleep. Sleep spindles in cortical channels are marked by black lines. Channel assignment as in Figure 2.

**FIGURE 4:**
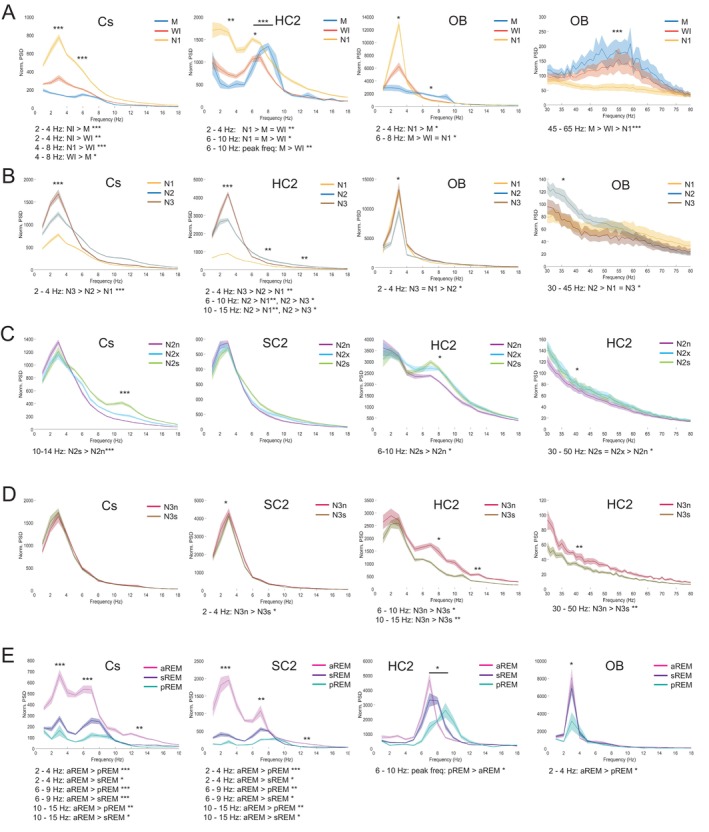
Mean power spectral density (PSD) plots of one 3‐h sleep session of eight mice in select channels and sleep (sub)stages. (A) Comparison between movement (M) and waking immobility (WI) and N1 sleep. (B) Comparison between N1, N2 and N3 stages. (C) Comparison between the N2 substages N2n, N2x and N2s. (D) Comparison between the N3 substages N3n and N3s. (E) Comparison between three REM sleep substages. Channels: Cs, skull electrode above the frontal cortex; HC2, middle channels in the triplet electrode in the hippocampus; OB, deeper channel of the doublet wire electrode in the olfactory bulb; SC2, middle channel in the triplet wire electrode in the S1 cortex. The graphs show the mean ± SEM of all eight mice. Within‐subjects pairwise comparisons (Nemenyi–Wilcoxon–Wilcox test) were run for the frequency bands around identified peak PSDs. The results are shown in the graph above the corresponding frequency band and more in detail below each graph. *p < 0.05, **p < 0.01, ***p < 0.001. Underlined symbols refer to significant differences in peak frequencies.

In a careful analysis of three successful (> 1 h of sleep) 3‐h multichannel recordings of 12 mice, we characterised nine sleep (sub)stages based on transitions of key electrophysiological signatures. We considered only (sub)stages that lasted 3 s or longer. The raw EEG signal of each transition is shown in Figure 2 for NREM (sub)stages and in Figure 3 for REM substages. The corresponding changes in the PSD are illustrated in Figure 4.

### 3.1 WI—N1 Transition

WI—N1 transition was identified by the disappearance of EMG bursts and the appearance of 1–2 s bouts of asymmetric 6–8 Hz oscillations in the frontal screw channels (Figures 2A and 4A), which may be analogous to frontocentral theta trains in human EEG during drowsiness (Schomer and da Lopes Silva 2018, 217). However, a much more striking change was the disappearance of prominent 45–65 Hz gamma oscillation in the OB (Figures 2A and 4A) as described earlier (Bagur et al. 2018). The change may be difficult to see within the usual 10‐s reading window of the raw EEG, but it really pops up in the PSD (Figure 2A). In fact, with the optimal placement of a single OB electrode (under the mitral cell layer) with a distant reference, one can tell whether the mouse is awake or asleep. In addition, the breathing‐associated prominent ~3 Hz OB oscillation became regular. The changes in the OB electrophysiology upon falling asleep most probably result from cessation of sniffing and odour processing, which are replaced by breathing at a regular pace around 3 Hz. Furthermore, in the hippocampal channel, one can see 1–2 s bouts of 5–7 Hz theta activity with superimposed gamma oscillation during occasional head movement during wake immobility while large irregular activity dominates the N1 sleep (Figure 2A). Remarkably, we never saw WI proceed directly to N2 sleep, but it was followed first by N1 sleep (Table 1).

**TABLE 1:** Transitions to and from waking immobility (WI) and non‐REM 1 (N1) sleep.

Transition	*N*	%	Transition	*N*	%
WI—M	98	19.0	M—WI	185	35.8
WI—N1	419	81.0	N1—WI	265	51.3
WI—N2n	0	0	N2n—WI	37	7.2
WI—N2x	0	0	N2x—WI	3	0.6
WI—N2s	0	0	N2s—WI	7	1.4
WI—N3n	0	0	N3n—WI	5	1.0
WI—N3s			N3s—WI	14	2.7
WI—aREM	0	0	aREM—WI	1	0.2
WI—all	517	100	to WI—all	517	100
N1—M	81	6.7	M—N1	0	0
N1—WI	265	21.8	WI—N1	419	34.4
N1—N2n	689	56.6	N2n—N1	358	29.4
N1—N2x	107	8.8	N2x—N1	59	4.8
N1—N2s	10	0.8	N2s—N1	36	3.0
N1—N3n	62	5.1	N3n—N1	98	8.1
N1—N3s			N3s—N1	167	13.7
N1—aREM	3	0.2	aREM—N1	32	2.6
N1—sREM	0	0	sREM—N1	48	3.9
N1—all	1217	100	to N1—all	1217	100

### 3.2 N1—N2 Transition

The N2 sleep was characterised by the appearance of cortical slow waves and 10–15 Hz sinusoidal sleep spindles of 0.5–1.5 s duration (Figures 2B and 4B) as in the human sleep EEG (Berry et al. 2015; Schomer and da Lopes Silva 2018). The slow waves were hard to detect on the skull EEG channel where they showed up as sharp negative deflections of variable amplitudes. However, intracortical electrodes below the granular cell layer 4 revealed the slow waves as prominent true surface positive round deflections of 150–300 ms in duration as shown earlier by full laminar recordings (Kahn et al. 2022, Figure 1d). Importantly, variation in amplitude and duration between the three intracortical channels reveals their local generation (Figure 2B) as opposed to other deflections that are generated distally. In fact, the slow waves recorded below layer 4 in the mouse somatosensory cortex ostensibly resemble the appearance of true surface negative (but displayed as upward deflections in standard clinical EEG) slow oscillations or K‐complexes of human EEG. As in human sleep EEG, the sleep spindles in mice have been shown to be local events (Kim et al. 2015). We could verify this observation. The cortical screw channel above the motor cortex and the intracortical channels in S1 cortex were likely to both show spindles in the same 10‐s window, but not necessarily exactly at the same time (Figure 2E,F).

### 3.3 Three N2 Substages

We observed N2 to alternate between three substages: one with slow waves without spindles (N2n), one with slow waves and clearly identified spindles (N2s) and a third substage without spindles but increased 10–15 Hz sigma(spindle) power in the background (N2x) (Figure 2D,E). The PSD of these three substages did not differ in delta power but did show higher sigma power for N2s than N2n on the frontal skull channel (Figure 4C). In addition, the hippocampal channels displayed bursts of large‐amplitude irregular activity at theta and low spindle frequency and increased gamma power during N2s and N2x (Figure 4C). Most often, N2x was an intermediary step between N2n and N2s (Figure 2D,E; Table 2), and its only statistical difference from N2n was the increase in gamma power (Figure 4C). Therefore, it remains questionable if it deserves assignment as a substage of its own.

**TABLE 2:** Transitions to and from non‐REM 2 (N2) sleep.

Transition	*N*	%	Transition	*N*	%
N2n—M	5	0.4	M—N2n	0	0
N2n—WI	37	3.0	WI—N2n	0	0
N2n—N1	358	29.1	N1—N2n	688	55.9
N2n—N2x	508	41.3	N2x—N2n	147	12.0
N2n—N2s	144	11.7	N2s—N2n	125	10.2
N2n—N3n	172	14.0	N3n—N2n	169	13.7
N2n—N3s			N3s—N2n	91	7.4
N2n—aREM	6	0.5	aREM—N2n	10	0.8
N2n—all	1230	100	to N2n—all	1230	100
N2x—WI	3	0.5	WI—N2x	0	0
N2x—N1	59	9.0	N1—N2x	107	16.3
N2x—N2n	147	22.3	N2n—N2x	508	77.2
N2x—N2s	375	57.0	N2s—N2x	6	0.9
N2x—N3n	70	10.6	N3n—N2x	33	5.0
N2x—N3s			N3s—N2x	4	0.6
N2x—aREM	4	0.6	aREM—N2x	0	0
N2x—all	658	100	to N2x—all	658	100
N2s—WI	7	1.3	WI—N2s	0	0
N2s—N1	36	6.7	N1—N2s	10	1.9
N2s—N2n	125	23.1	N2n—N2s	144	26.7
N2s—N2x	6	1.1	N2x—N2s	375	69.4
N2s—N3n	0	0	N3n—N2s	4	0.7
N2s—N3s	278	51.5	N3s—N2s	2	0.4
N2s—aREM	88	16.3	aREM—N2s	5	0.9
N2s—all	540	100	to N2s—all	540	100

### 3.4 Two N3 Substages

As in humans, the N3 stage was characterised by a train of continuous 170–350 ms delta‐waves (our definition two or more per s) (Berry et al. 2015). The delta waves were present in all channels but not in full synchrony (Figure 2C). The transition from N2 to N3 was associated with an increase in delta power (2–4 Hz) and an abrupt decrease of hippocampal power in all frequencies above 5 Hz (Figures 2C,F and 4D). There were subtle but consistent differences in the transitions between N3 and N2n versus N3 and N2s, which made us consider that N3 has two substages, which we coined N3n and N3s, correspondingly. The classic N3n usually followed N2n sleep and returned to the N2n stage (Table 3). The transition was synchronous in all channels (Figure 2C). In contrast, N3s after N2s showed the onset of delta‐waves only 1–1.5 s after the abrupt cessation of hippocampal power over 5 Hz (Figure 2F). In addition, the drop in hippocampal power at theta, spindle and gamma bands was more robust in N3s than in N3n (Figure 4D). Whereas N3n most often returned to N2n, N3s almost never returned to N2s but had mostly a course toward N1 and microarousal (Table 3).

**TABLE 3:** Transitions to and from non‐REM 3 (N3) sleep.

Transition	*N*	%	Transition	*N*	%
N3n—WI	5	1.6	WI—N3n	0	0
N3n—N1	98	31.7	N1—N3n	62	20.1
N3n—N2n	169	54.7	N2n—N3n	172	55.7
N3n—N2x	33	10.7	N2x—N3n	70	22.7
N3n—N2s	4	1.3	N2s—N3n		
N3n—aREM	0	0	aREM—N3n	5	1.6
N3n—all	309	100	to N3n—all	309	100
N3s—WI	14	5.0	WI—N3s		
N3s—N1	167	60.1	N1—N3s		
N3s—N2n	91	32.7	N2n—N3s		
N3s—N2x	4	1.4	N2x—N3s		
N3s—N2s	2	0.7	N2s—N3s	278	100
N3s—all	278	100	to N3p—all	278	100

### 3.5 Three REM Substages

It is well known that the onset of REM sleep in rodents is not easy to define. Gottesman was the first to coin the term ‘intermediate stage’ in the 60's, (Gottesmann 1996) describing a substage between NREM and REM where the rat hippocampus expressed regular theta activity while the cortical EEG showed increased spindle activity. We agree with this observation. The REM epoch always followed N2s sleep, before the onset of regular theta activity in the hippocampus. We defined the onset of REM when all electrodes in one hippocampus showed synchronised sinusoidal 6–8 Hz theta activity while the EMG shows low amplitude. Typically, the REM continued for 10–30 s so that the cortical channels showed an increasing amount of theta activity in the background yet continued to display slow waves and/or spindles. At the same time, synchronised theta was present on all hippocampal channels (Figure 3A,B). We call this phase of REM sleep asynchronous REM (aREM). Often the REM onset looked like starting a car with an old battery on a frosty morning: after a series of failed attempts to start, the REM was eventually established. One could detect short bouts of less regular theta in the hippocampus preceding the constant local theta (Figure 3A) or such that died out and did not lead to REM at all (Figure 3E). If REM continued further, theta activity evaded all cortical channels. We call this latter phase, which could last for a few minutes, synchronous REM (sREM Figure 3B). The sREM was characterised by a stable theta frequency (6–8 Hz). However, at times it was interrupted by bouts of faster and slower theta synchronised with the cortical channels (Figure 3D). This phasic REM (pREM) has been linked to eye movements in rodents and is analogous to human REM (Brankačk et al. 2012). The three REM substages can also be distinguished by their PSD profiles. The aREM has much stronger delta, low theta and sigma power than sREM and pREM in the cortical channels (Figure 4E), whereas the pREM can be separated from sREM by its broader theta power indicating variable frequency and a higher peak power (Figure 4E) and weaker delta (2–4 Hz) power in the OB (Figure 4E). Notably, the OB channel is the only one that does not synchronise with the hippocampal theta at any REM substage. In contrast to the gradual onset, the sREM usually ended suddenly, with or without a brief bout of a second aREM, into N1 or microarousal (Figure 3C; Table 4).

**TABLE 4:** Transitions to and from REM sleep.

Transition	*N*	%	Transition	*N*	%
aREM—WI	1	0.9	N1—aREM	3	2.8
aREM—N1	32	30.2	N2n—aREM	6	5.7
aREM—N2n	10	9.4	N2x—aREM	4	3.8
aREM—N2s	5	4.7	N2s—aREM	88	83.0
aREM—N3	5	4.7	N3—aREM	0	0
aREM—sREM	53	50.0	sREM—aREM	5	4.7
aREM—all	106	100	to aREM—all	106	100
pREM—sREM	107	100	sREM—pREM	107	100
pREM—all	107	100	to pREM—all	107	100
sREM—N1	48	30.0	N1—sREM	0	0
sREM—aREM	5	3.1	aREM—sREM	53	33.1
sREM—pREM	107	66.9	pREM—sREM	107	66.9
sREM—all	160	100	to sREM—all	160	100

### 3.6 Succession of Sleep Stages

Next, we analysed how the identified sleep (sub)stages followed each other during a well‐slept 3‐h recording session in eight mice. The results are summarised in Tables 1, 2, 3, 4. The sleep sequence always started with N1. If not leading to arousal, it continued to the N2n stage in 80% of the cases. N2n returned to N1 in 29% but continued with N2x or directly with N2s in 53% of the cases. It could also lead to N3 (14%). N2x most often (57%) led to N2s, which ended with N3p in 52% of the cases and led to either N1 or N2n. About 16% of N2s epochs led to aREM and further to sREM/pREM. Looking backward, 88% of aREM epoch were preceded by N2s and a further 4% by N2x. So, it looks like the mouse sleep consists of a few sequences that repeat periodically.

### 3.7 Mouse NREM Sleep Is Modulated by an Infra‐Slow Oscillation

**FIGURE 5:**
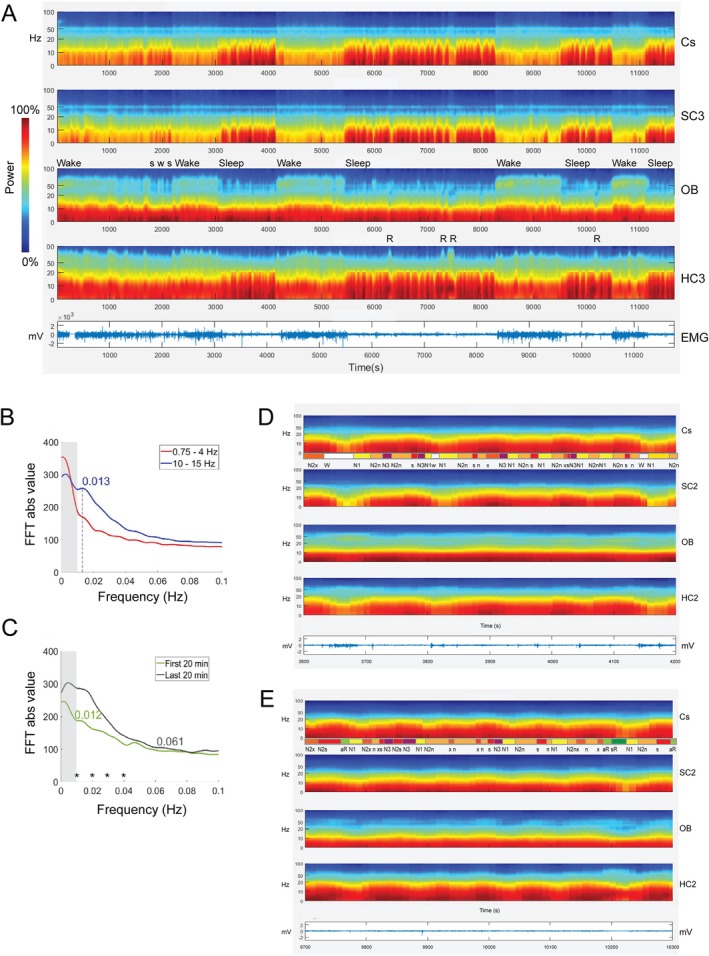
Infra‐slow oscillation (ISO) during NREM sleep. (A) PSD plot of the entire 3‐h recording session of Mouse 30. Sleep epochs show a striking candle flame‐like pattern in the cortical and hippocampal channels, which reflect an underlying ISO via a phase‐amplitude coupling of frequencies up to 25 Hz. These are interrupted by wake, arousal and REM (R) epochs. Channel assignment as in Figure 4, but SC3 and HC3 refer to the deepest channel in the triplet electrode. (B) Mean absolute values of a Fast Fourier analysis of the ISO across all 12 mice between 0.01 and 0.1 Hz in delta and sigma bands. The sigma band shows a small peak at 0.013 Hz. (C) The same analysis separated into the first 20 min and the last 20 min of the 3‐h recording. The ISO is significantly stronger during the last 20 min between 0.01 and 0.04 Hz. (D) A 10 ‐min excerpt of the PSD plot of Mouse 124 during the early third of the recording. The ISO appears irregular. Different sleep (sub)stages are marked with letters and colour codes between the cortical channels Cs and SC2. Wake (W,w) and N1 are positioned around the weak ISO nodes, while N2x, N2s (x, s) and N3 are located on the strong nodes. (E) A 10‐min excerpt of Mouse 32 PSD plot during the last third of the recording. The ISO appears to have several and more regular cycles. Note the location of aREM (aR) and sREM (sR) epochs on the declining slope of the ISO.

When we first viewed the PSD distribution during the entire 3‐h sleep recording, the first observation that caught the eye was the palisade of flame‐like patterns in all channels during sleep epochs (Figure 5A). It looked like the power in a broad frequency band fluctuates periodically. Importantly, this is not a sampling artefact (we used 10‐s bins in calculating the PSD), since changing the bin width to 4 s did not change the pattern. These observations concurred with recent observations that the EEG power in a broad frequency range (~5–30 Hz) is modulated by an infra‐slow oscillator in a way that resembles phase—amplitude coupling (Lecci et al. 2017; Kjaerby et al. 2022).

Next, we set out to determine the frequency of this putative infra‐slow oscillation (ISO). To this end, we calculated FFT on the spindle power peaks as described earlier (Lecci et al. 2017). When averaging over all sessions and all 11 mice, we found an ISO frequency peak at around 0.013 Hz (Figure 5B). However, there were significant variations between the exact peak frequency among individual mice (Figure S2). In addition, we assessed a possible shift in the ISO frequency during accumulated sleep. This was done by comparing the first 20 min and last 20 min of the daily 3‐h recording sessions (but only for those with > 1 h of sleep). The power of the ISO was significantly higher between 0.01 and 0.04 Hz during late sleep compared to early sleep (Figure 5C). However, there was no consistent shift in the ISO peak between early and late sleep. The irregularity of the ISO can also be seen when looking at the PSD fluctuation at a shorter time scale (Figure 5D,E). It looks like we are not dealing with a neural oscillator but rather with quasi‐periodic signal fluctuations within certain frequency limits.

At the next stage, we compared the relationship between the PSD fluctuations and the characterised signature of the nine identified sleep (sub)stages. Figure 5D shows this comparison for an early part of the recorded sleep and Figure 5E for a late part. The excerpt of early sleep does not contain any REM epoch and includes moments of waking immobility. In contrast, late sleep has frequent REM epochs but seldom any arousals. A clear periodicity can be seen in both excerpts. The peaks of the PSD power contain N2s and N3s epochs, whereas the troughs contain N1 and REM (shortly aREM or sREM) epochs. The ascending slope usually contains the N1—N2n—N2x/N2s sequence, whereas the descending slope typically contains an N2x/N2s—N3s—N1 or N2x/N2s—aREM—(sREM)—N1 sequence. Thus, it seems that the sleep stages follow the putative underlying ISO.

**FIGURE 6:**
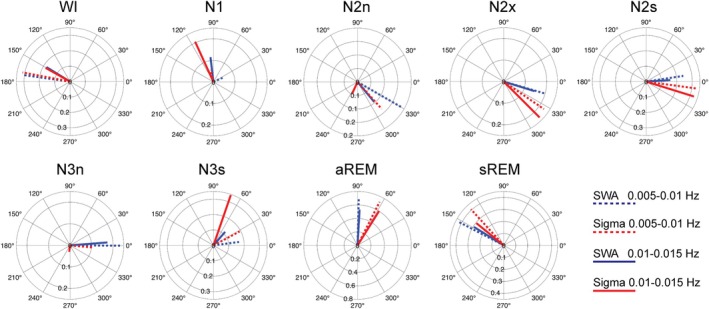
Phase preference of the start of different sleep substages on the underlying ISO. The phase preference is shown by the phase and length of the average vector of all stage transitions of a representative sleep recording session of eight mice. The vector was calculated separately for the delta/slow‐wave (SWA, blue) and sigma (red) bands. In addition, the ISO was divided into lower (0.005–0.01 Hz, stippled line) and higher (0.01–0.015 Hz, solid line) range. The strength of the average vector is indicated by the scale from 0 to 1 on the concentric rings. The orientation of the phases corresponds to the ISO power, such that 90° represents the ISO peak and 270° its trough. Note the consistent counterclockwise rotation of the phase in the sequence N2n—N2x—N2s—aREM—sREM. There is also a consistent 20°–30° advance of the SWA‐based phase compared to sigma‐based phase in these sleep stages. For WI and N1 the rare but robust transitions from sREM are not included.

To quantify these observations, we analysed whether the onset of identified sleep (sub)stages (the transitions) falls on a special phase of the ISO. Based on the observed ISOs in our dataset, we focused this analysis on ISO between 0.005 and 0.015 Hz. We band‐pass filtered the FFT values of delta/slow‐wave (0.75–4 Hz) and sigma (10–15 Hz) bands of all channels and calculated the phase of ISO (delta and sigma separately) at every sleep‐stage transition. Each transition to a specific sleep stage was represented by a unit vector with a specific phase. Next, we calculated the mean phase vector per mouse per substage, composed of the mean starting phase of all epochs. This was normalised by the number of epochs to yield a length between 0 (no phase preference) and 1 (all epochs start with the same ISO phase) and the average phase. As the last step, phase vectors of all eight mice were averaged for each sleep substage. The results are summarised in Figure 6. The strength of this phase coupling in terms of the average vector length ranged from 0.2 of N1 to 0.7 of aREM. When the length was 0.3 or more, the lower (0.005–0.01 Hz) and higher (0.01–0.015 Hz) ISO bands yielded the same estimate suggesting that the ISO covers a broad frequency range. In contrast, the phase based on delta versus sigma bands differed so that the delta‐based estimate was about 20° more advanced in the counterclockwise direction for most sleep substages. There was a clear progression in the ISO phase in the counterclockwise direction with N2n co‐occurring at a low power (270°), N2s halfway on the rise (0°), aREM or N3s near the peak (90°) and sREM on the descending phase (130°–150°). N3s, an alternate substage for N2s to proceed, shared the same phase as aREM (Figure 6).

### 3.8 Role of Central Noradrenergic System in Regulating the PSD Fluctuation

Several recent reports have provided evidence that the mouse sleep stages are under the control of the broad noradrenergic projections from the LC to the forebrain. The sleep spindle generation in the thalamus can be induced or suppressed by optogenetic manipulation of LC (Osorio‐Forero et al. 2021). Moreover, the frontal sigma power fluctuates at around 0.02 Hz with frontal cortical NA levels (Kjaerby et al. 2022). To test whether ISO at a broader brain context depends on LC NA, we performed two manipulations of the systems in five additional mice and continued our multichannel sleep recordings.

**FIGURE 7:**
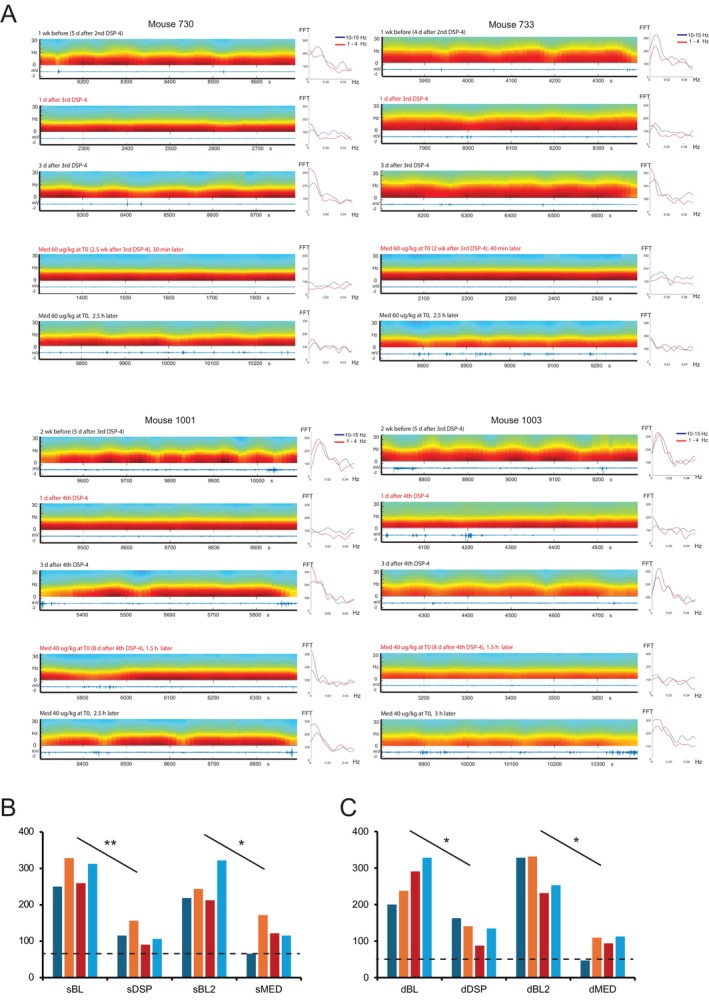
(A) Heatmap PSD plots (cortical screw channel + EMG) of a representative 10‐min epoch judged as deep sleep based on the flat EMG of Mice 730, 733, 1001 and 1003. Top row: Before DSP‐4; second row: Day after the third or fourth DSP‐4 injection; third row: Recovery 3 days later; fourth row: Shortly (30–90 min) after the medetomidine injection; bottom row: Partial recovery 2.5–3 h after the injection. The FFT plots on the right of the heatmaps were generated the same way as described in Figure 1. The delta (0.75–4 Hz, red) and sigma (10–15 Hz, blue) periodicity around 0.01 Hz attenuate drastically after both DSP‐4 and medetomidine treatments. A full recovery of this ISO periodicity is seen 3 days after the effective DSP‐4 injection, whereas partial recovery after the medetomidine administration is seen already 2.5 h after the injection. (B) Peak sigma power in individual mice (in numerical order) before (sBL) and after (sDSP) DSP‐4 injection and before (sBL2) and after (sMED) medetomidine injection. (C) The same for delta (d) peak power. The dashed line indicates noise level power in the corresponding frequency band. **p < 0.01, *p < 0.05, paired‐sample *t* test.

**FIGURE 8:**
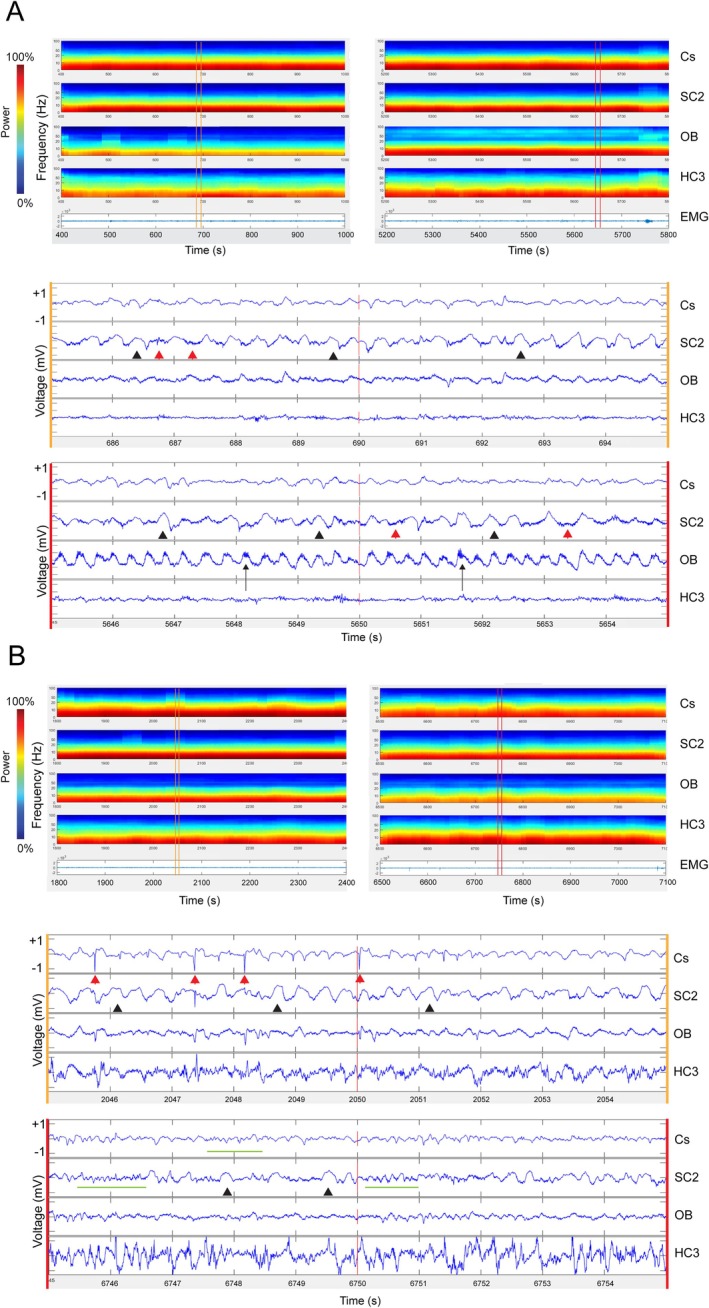
(A) Top: Heatmap PSD plots of two 10‐min epochs of immobility of Mouse 1001 a day after the fourth DSP‐4 injection. There is no indication of ISO fluctuation during these epochs. Bottom: 10‐s EEG excerpts of the epochs indicated with double orange and red lines. The EEG shows continuous N3‐like delta‐waves in the intracortical channel (SC2), with discernible down‐states (suppression of neuronal firing, black triangles) and up‐states with fast activity (red triangles). Based on the OB signal, breathing is slow and irregular during the first EEG excerpt but typical regular N1‐like during the second excerpt with atypical high‐gamma oscillation (arrows). Channels: Cs, cortical screw; HC3, deep hippocampal channel; OB, olfactory bulb; SC2, middle intracortical channel. (B) Top: Heatmap PSD plots of two 10‐min epochs of immobility of Mouse 732 taken 30 min and 2 h after medetomidine (60 μg/kg) injection. (Bottom) 10‐s EEG excerpts of the epochs indicated with double orange and red lines. The cortical ISO fluctuation is completely missing during the first 45 min but then gradually recovers. The ostensible fluctuation in the sigma frequency in the skull (Cs) channel proved to arise from frequent spiking (red triangles) in a closer examination of the EEG during the earlier epoch. Otherwise, the intracortical channel (SC2) shows continuous N3‐like delta‐waves (black triangles) as in the recording after DSP‐4 in Figure 7A. During the latter immobility epoch one can see typical N2s sleep with delta‐waves and spindles (green horizontal lines). Channel assignments as in Figure 8A.

First, we used the old LC selective adrenergic neurotoxin DSP‐4 to damage noradrenergic axons. The most common administration regimen, two injections at 50 mg/kg with 3 intervening days, did not affect the ISO in any of the mice. However, when there were 3 weeks after the previous injection (either third or fourth), the ISO disappeared completely in the recording done 1 day after the fourth injection in four mice (Figure 7). The intracortical EEG in these mice showed continuous delta waves at about 2 Hz as if the sleep was locked into the N2n or N3n stage (Figure 8A). However, in one mouse, the ISO only slowed down. In all mice, the acute behavioural effect of DSP‐4 was nevertheless similar and robust. The mouse stood motionless in the corner of the cage and looked agitated for about half an hour.

Additional injections up to 4 after the first DSP‐4 administration do not kill further LC neurons, and the 3 weeks is too short a time for the noradrenergic axons to regenerate (Markussen et al. 2023). In line with these observations, we found a substantial depletion of noradrenergic fibres in the parietal cortex in all mice when perfused 4 weeks after the last DSP‐4 injection (Figure S3). However, rapid recovery of LC neurotransmission is suggested to happen via increased neurotransmitter release from the preserved axon terminal and postsynaptic supersensitivity (Harro et al. 1999). Therefore, we challenged the LC system further by systemic administration of the selective α2‐adrenoceptor agonist medetomidine. A single injection of medetomidine at a subanesthetic 40–60 μg/kg dose before the recording onset suppressed the ISO for 30–60 min in all five injected mice (Figure 7). Similarly to DSP‐4, the intracortical EEG initially displayed continuous 2–3 Hz delta‐waves, while the skull EEG showed a periodic ~5 Hz spike–wave pattern (Figure 8B). However, a normal sleep EEG with ISO and sleep spindles, but without any REM epoch, returned in the latter part of the recording (Figure 8B). This is compatible with the short tissue half‐life of medetomidine (Salonen 1989).

Together, these two drug manipulations support the crucial role of LC noradrenergic projections underlying the ISO‐like fluctuation of electrical brain activity during sleep.

## 4 Discussion

The present study addressed the question of whether we could refine the mouse sleep staging by identifying characteristics electrophysiological signatures of each stage rather than calculating ratios of arbitrarily defined frequency bands. To this end, in addition to the conventional recording of skull EEG and neck EMG, we utilised 11 recording channels, including an intracortical triplet electrode, two hippocampal triplet electrodes, and a doublet electrode in the OB. The inclusion of these channels greatly helped detect the cortical slow waves and the wake–sleep boundary, respectively. This way, we could recognise all four established human sleep stages N1, N2, N3 and REM in the mouse. In addition, we could identify three N2 substages (N2n, N2s and N2x), two N3 substages (N3n and N3s) and three REM substages (aREM, sREM and pREM). The characteristics of these sleep stages are summarised in Table 5.

**TABLE 5:** Summary of characteristic EEG/EMG signatures at different recording locations during each sleep substage in the mouse.

Sleep stage	CX_skull	CX_intracx	OB	HC	EMG
WI	Low V	Low V	45–65 Hz + irregular ~3 Hz	6–8 Hz theta at times	Bursts of high V
N1	6–8 Hz bouts	Low V	Regular ~3 Hz, breathing	Large irregular activity = LIA	Medium V
N2n	Single slow waves, low V	Single slow waves, high V	~3 Hz less regular	LIA	Low V
N2x	Single slow waves, low V	Single slow waves, high V	~3 Hz less regular	Bouts of 10–15 Hz + LIA + gamma	Low V
N2s	10–15 Hz spindles	10–15 Hz spindles	~3 Hz less regular	Bouts of 10–15 Hz + LIA + gamma	Low V
N3n	Trains of delta waves 2–4 Hz	Trains of delta waves 2–4 Hz	~3 Hz less regular	Trains of delta waves + LIA	Low V
N3s	Trains of delta waves 2–4 Hz	Trains of delta waves 2–4 Hz	~3 Hz less regular	Trains of delta waves only	Low V
aREM	Like N2s	Like N2s	~3 Hz less regular	6–8 Hz theta, regular	Very low V
sREM	6–8 Hz theta, regular	6–8 Hz theta, regular	~3 Hz less regular	6–8 Hz theta, regular	Very low V
pREM	5–9 Hz theta, variable	5–9 Hz theta, variable	~3 Hz less regular	5–9 Hz theta, variable	Very low V

Dividing the mouse N2 sleep into N2n and N2s substages based on the absence or presence of spindles deviates from the established staging of human sleep. However, it is not as radical as it sounds since the human N2 includes epochs without spindles and epochs with clusters of spindles. The justification for this distinction is that the generation of cortical slow waves and spindles involves different neural networks both in the neocortex (Niethard et al. 2018) and thalamus (Bandarabadi et al. 2020). Furthermore, apart from showing that the cortico‐thalamic circuit displays a different stage during spindles from other N2 sleep, we demonstrated that the hippocampal EEG also differs clearly between these two stages, with prominent delta waves during N2n and sigma frequencies popping up during N2s. On the other hand, the boundary between the N2n and the onset of N2s is less clear. Usually, one first sees a change in the background, especially in the hippocampal channels, and the appearance of an irregular burst of sigma frequencies in the cortical channel but not the clean sinusoidal spindles. Therefore, we defined the duration of N2s from the first to the last identified clean spindle and assigned the transition stage to a separate substage N2x. Statistically, these substages all differ in their frequency composition, but their utility in the long run remains to be seen.

We are not aware of any published journal article that describes N3 sleep in the mouse except an extensive study reported in bioRxiv only (Lacroix et al. 2018). We did observe bursts of delta waves that were most distinctive in the intracortical channel below layer 4 (Figure 2C). We considered delta waves a burst when we saw two waves every second for at least 3 s. Typically, these lasted only 3–5 s, seldom over 10 s. This is not essentially different from human N3, which also often shows short (< 5 s) bursts of delta waves in combination with longer bursts. The N3 definition according to the AASM criteria (delta bursts occupying 20% time within a 30 s epoch; (Berry et al. 2015; Schomer and da Lopes Silva 2018)) is statistical and, without any doubt, sufficient for clinical purposes. However, if we want to relate firing changes in a certain population of neurons in a deep nucleus with the cortical EEG change, one should match them with the delta bursts themselves rather than a period with an increased likelihood of occurrence. The N3 epoch typically occurred after the N2 stage. However, when an N3 epoch occurred after the N2n stage, it usually also returned to the N2n substage. In contrast, when it followed the N2s stage, it hardly ever returned to that substage but proceeded to N1 and a microarousal. Since the hippocampal activity upon N3 onset also differed (earlier onset and more robust attenuation of all but delta frequencies) after the N2s, we consider this a separate substage and coined it correspondingly N3s (in contrast to conventional N3n that follows N2n stage). This kind of sudden drop in hippocampal EEG signal preceding microarousal in the mouse has been described earlier (Dos Santos Lima et al. 2019) but not associated with a separate sleep substage.

We divided the mouse REM sleep first into asynchronous (aREM) and synchronous (sREM) REM. Our aREM corresponds to the old concept of intermediate stage sleep and concurs with the idea that the REM sleep gradually progresses from the brainstem to the cortex (Gottesmann 1996). However, we do not fully agree with the contention that aREM is limited to the state when spindles are prominent in the cortex while the hippocampus shows theta activity (Gottesmann 1996). According to our data, the cortical channels can often display only slow waves corresponding to the N2n stage when the hippocampus shows regular theta activity. There is a growing consensus that thalamo‐cortical spindles precede REM sleep and are a prerequisite for the transition to REM sleep (Bandarabadi 2020; Kjaerby et al. 2022). This corresponds to our observations as well. Namely, in our data, the N2s substage always preceded the aREM state. Further support for the idea of gradual onset of REM comes from the observations of unsuccessful REM starts (Figure 3E). Sometimes several momentary theta bursts in only one hippocampal channel were seen before all channels synchronised. These ‘cold’ starts could proceed to stable aREM and further to sREM but also end all of a sudden at the aREM stage. The established sREM was frequently intermingled with periods where the theta frequency waxed and waned. This type of REM has been coined pREM in the earlier literature and associated with eye movements (Brankačk et al. 2012). Notably, the REM detection and identification of REM substages are heavily dependent on available hippocampal recordings. A skull or epidural EEG will therefore robustly underestimate the REM duration as they totally miss the aREM phase.

The most important new finding in the present study was that the mouse sleep stages are organised sequentially on top of an infra‐slow fluctuation (ISO) of the EEG power in a broad frequency band, roughly 1–30 Hz but most prominent within the delta (1–4 Hz) and the spindle frequency or sigma band (10–15 Hz). When averaged over all mice and sessions, we obtained an estimate of 0.013 Hz as the ISO frequency, although it clearly varied between individual mice and on a moment‐to‐moment basis during the recording even in a single mouse, which supports the claim of quasi‐periodic ISO (Thompson et al. 2014; Kropotov 2022). An ISO of 0.025 Hz has earlier been described to account for fluctuating excitability of the hippocampal network in rats under urethane anaesthesia (Penttonen et al. 1999). Another earlier study in mice also revealed that the sigma (spindle) power during NREM sleep oscillated around 0.02 Hz, and that this oscillation was most robust in the somatosensory cortex (Lecci et al. 2017). A similar modulation was found in the heart rate and in synchrony with the EEG sigma power. Moreover, such an ISO around 0.02 Hz underlying NREM fluctuations was described also in the conventional human EEG (Lecci et al. 2017) and earlier more directly with direct current scalp EEG within the range of 0.02–0.2 Hz (Vanhatalo et al. 2004). These observations probably all describe the same phenomenon, although the estimates of its frequency vary a bit between the studies.

The source of this ISO that organises the mouse sleep pattern is currently under intensive investigation. The observation that the ISO in the OB is in phase with cortical and hippocampal channels excludes the thalamus as the primary source of this ISO, since to our knowledge the OB has not been shown to receive thalamic input. In addition, the fact that the heart rate variability also follows the same oscillation indicates that the autonomic nervous system is paced with the same oscillating circuitry as the hippocampus or the neocortex (Lecci et al. 2017). Further, the finding that the cortical spindle frequency is modulated at around 0.02 Hz during NREM epochs in humans and mice rules out the contribution of breathing as the underlying factor, since the breathing rate in NREM sleep varies from 0.2 Hz in humans to 3–4 Hz in mice.

Two recent reports during mouse sleep strongly suggest that the observed ISO underlying the cortical fluctuation of spindle power reflects the activity of LC. Osorio‐Forero et al. (2021) first blocked noradrenergic α1‐ and β‐adrenoceptors unilaterally in the ventral posterior thalamus of mice and observed the disappearance of the 0.02 Hz ISO of sigma power. Then they optogenetically stimulated or inhibited LC. Notably, stimulation of LC when spindle power started rising suppressed the 0.02 Hz ISO. Conversely, LC inhibition during the declining phase of spindle power prolonged spindle activity. Kjaerby et al. (2022) extended these findings by monitoring NA levels in the mouse frontal cortex with a fluorescent biosensor and LC activity with Ca^2+^ imaging. LC activity occurred in bursts about every 30 s and correlated with rapidly ascending NA levels in the frontal cortex. Between the bursts, NA levels slowly declined with a relatively constant slope. Sleep spindles occurred during the descending phase of NA levels. Intriguingly, LC bursts and the corresponding ascent in NA levels after a short NA decline resulted in a microarousal, whereas a NA rise after a longer decline resulted in awakening. If the NA levels continued to decline even longer, about 40 s, a transition to REM sleep occurred. REM sleep was terminated by a rapid increase in NA levels again. So, it appears that the occurrence of sleep spindles (high power in the 10–15 Hz band), microarousals, and REM sleep are all largely in pace with the bursting firing pattern of LC neurons and the resulting fluctuations in thalamic and cortical NA levels in mouse sleep. Our finding of sleep substage occurrence at certain phases of the underlying ISO is in line with these findings. Typically, N1 and N2n stages were found on the ascending ISO slope, N3n, N2x and N2s substages on the top while N3s and all REM substages were located on the descending slope (Figure 5D,E). Or correspondingly, when we analysed the onset of each substage, N2n started around the ISO trough, N2x and N2s on the ascending phase at the 0 line, and N3s and aREM near the peak and sREM on the descending slope (Figure 6). If we convert these ISO phases to cortical NA levels according to the Kjaerby et al. study, N2n started after the NA levels had reached the peak, and N2s and aREM during the declining NA levels. If the NA decline lasted long enough, a sREM state ensued (Kjaerby et al. 2022).

We extended the observations of cortical ISO‐like spindle power fluctuation during NREM sleep synchronising with local fluctuations of NA levels by showing a causative role of LC in these fluctuations. We showed that damaging LC NA axons by the selective toxin DSP‐4 abolished the ISO‐like fluctuation of delta and spindle frequencies similarly in the cortex, hippocampus and OB. Notably, this happened only after the third or fourth DSP‐4 injection and was observed only during the following day of the injection (a complete recovery of ISO 3 days after the injection). These observations are fully in agreement with the extreme plasticity of the LC system (Markussen et al. 2023). Moreover, when the ISO had fully recovered, a subanesthetic dose of the selective α2‐adrenoceptor agonist, medetomidine, again abolished the ISO, but only for 30–45 min after the injection, which concurs with its short tissue half‐life. The α2‐adrenoceptors serve as autoreceptors at the body of LC neurons and axon terminals, which most likely explains the effect. However, there are also postsynaptic α2‐adrenoceptors at the LC innervation areas, which may also partly contribute to the effect. Anyway, both experiments lend strong support to the notion that the ISO is dependent on the functional LC NA projections.

If LC is the generator of ISO during sleep, what would explain the irregular nature of this oscillation or EEG power fluctuation? We measured an average frequency of this ISO around 0.013 Hz but noted substantial variation between mice and within even a single 3‐h sleep episode in individual mice. Typically, the fluctuation had a higher amplitude and shorter period toward the end of the 3‐h recording than in the beginning. Kjaerby and collaborators suggested that this ISO irregularity derives from varying lengths of the descending phases of cortical NA levels (Kjaerby et al. 2022). A recent study, (Silverman et al. 2025), suggests that the fluctuation of NA levels in the LC terminals stems from fatigue of NA neurons after prolonged firing, which explains the variable length of NA fluctuation cycles.

## 5 Conclusions

By complementing the common skull or epidural EEG channels with triple intracortical, double OB and triple intrahippocampal channels, we could identify EEG/LFP signatures that help separate the conventional N1, N2, N3, REM boundaries in mouse sleep and further identified putative N2, N3 and REM substages. Especially, the OB electrode proved to be instrumental in separating N1 sleep from waking immobility, while the intracortical electrodes greatly helped identify cortical delta/slow oscillation during N2–N3 sleep and the hippocampal electrodes identified N2 and N3 substates. The use of these additional electrodes can therefore be recommended for rodent sleep studies aiming to unveil mechanisms of sleep regulation. However, these data are difficult to translate to human sleep studies. One future step to this end, although technically challenging, would be a combination of these intracranial recordings with EOG.

We could show that the succession of sleep (sub)stages, at least in the mouse, is dependent on the phase of an underlying quasi‐periodic ISO‐like oscillation that disappears when the LC NA neurotransmission is compromised. Further studies will reveal if the human sleep stages also follow the fluctuating levels of cortical NA. There are already observations of a ~0.02 Hz ISO in the human EEG power during NREM sleep (Lecci et al. 2017; Vanhatalo et al. 2004). Thus, the NA fluctuation may prove to be the basic regulator of sleep microstructure and similar in duration in both mice and humans.

## Author Contributions

**Heikki Tanila:** conceptualization, funding acquisition, writing – original draft, writing – review and editing, project administration, supervision, resources.

## Funding

This work was supported by the Research Council of Finland (340377), the Jane and Aatos Erkko Foundation and the Finnish Cultural Foundation.

## Conflicts of Interest

The authors declare no conflicts of interest.

## Supporting information


**Figure S1:** Location of electrode tips based on electrolytic lesions and electrophysiological landmarks. (A) Mice in the main sleep staging study, (B) mice in the DSP‐4 and medetomidine study. The intracranial wire electrodes are shown individually in each mouse while the common setup of two recording bone screw electrodes and the ground and reference are shown on the bottom.

**Figure S2:** The Identified peaks of the infra‐slow oscillation (or power fluctuation) in individual mice calculated as shown in Figure 1. The blue line is based on power fluctuations in the sigma band (10–15 Hz) while the red line shows the fluctuation in the delta or slow‐wave (0.75–4 Hz). Significant peaks are shown with numbers. The shaded area indicates that the analysis was done for putative ISO frequences between 0.01 and 0.1 Hz.

**Figure S3:** Tyrosine hydroxylase staining with Ni‐DAB enhancement of the visual cortex in two control mice and four DSP‐4 (50 mg/kg i.p.) treated mice. The continuous line indicates the brain surface while the dashed line denotes the L1—L2 border. Note the dense meshwork of NA fibres that run mainly tangentially to the brain surface in layer 1 in the control mice. These have almost completely disappeared in the DSP‐4 treated mice. Instead, one can see individual fibres perpendicular to the brain surface (white arrows). These are probably regrowing axons of locus coeruleus NA neurons. Scale bar = 50 μm.

## Data Availability

The data that support the findings of this study are available on request from the corresponding author. The data are not publicly available due to privacy or ethical restrictions.
