# Supplementary figures and images for: Refined Division of Sleep Stages in the Mouse Based on Distributed Deep Electrodes and Underlying Infra‐Slow Oscillation
Source: J Sleep Res. 2025 Dec 15;35(4):e70262. doi: 10.1111/jsr.70262 (PMC13357931; doi:10.1111/jsr.70262)

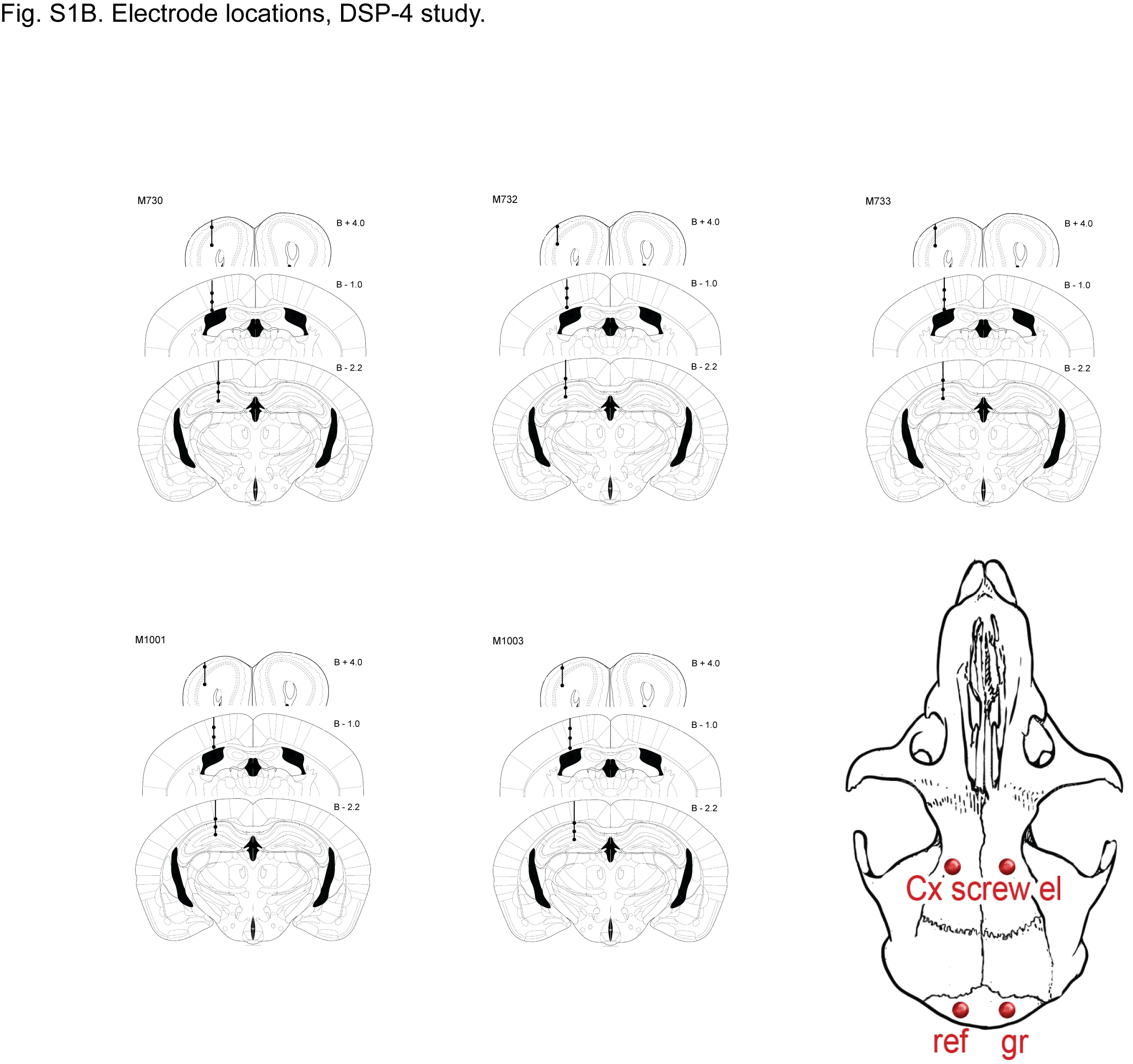

Supplement: Supplementary file 1 — Figure S1: Location of electrode tips based on electrolytic lesions and electrophysiological landmarks. (A) Mice in the main sleep staging study, (B) mice in the DSP‐4 and medetomidine study. The intracranial wire electrodes are shown individually in each mouse while the common setup of two recording bone screw electrodes and the ground and reference are shown on the bottom. [file JSR-35-e70262-s001.zip › jsr70262-sup-0002-FigureS1@Fig. S1B Electrode locations DSP-4.tif]

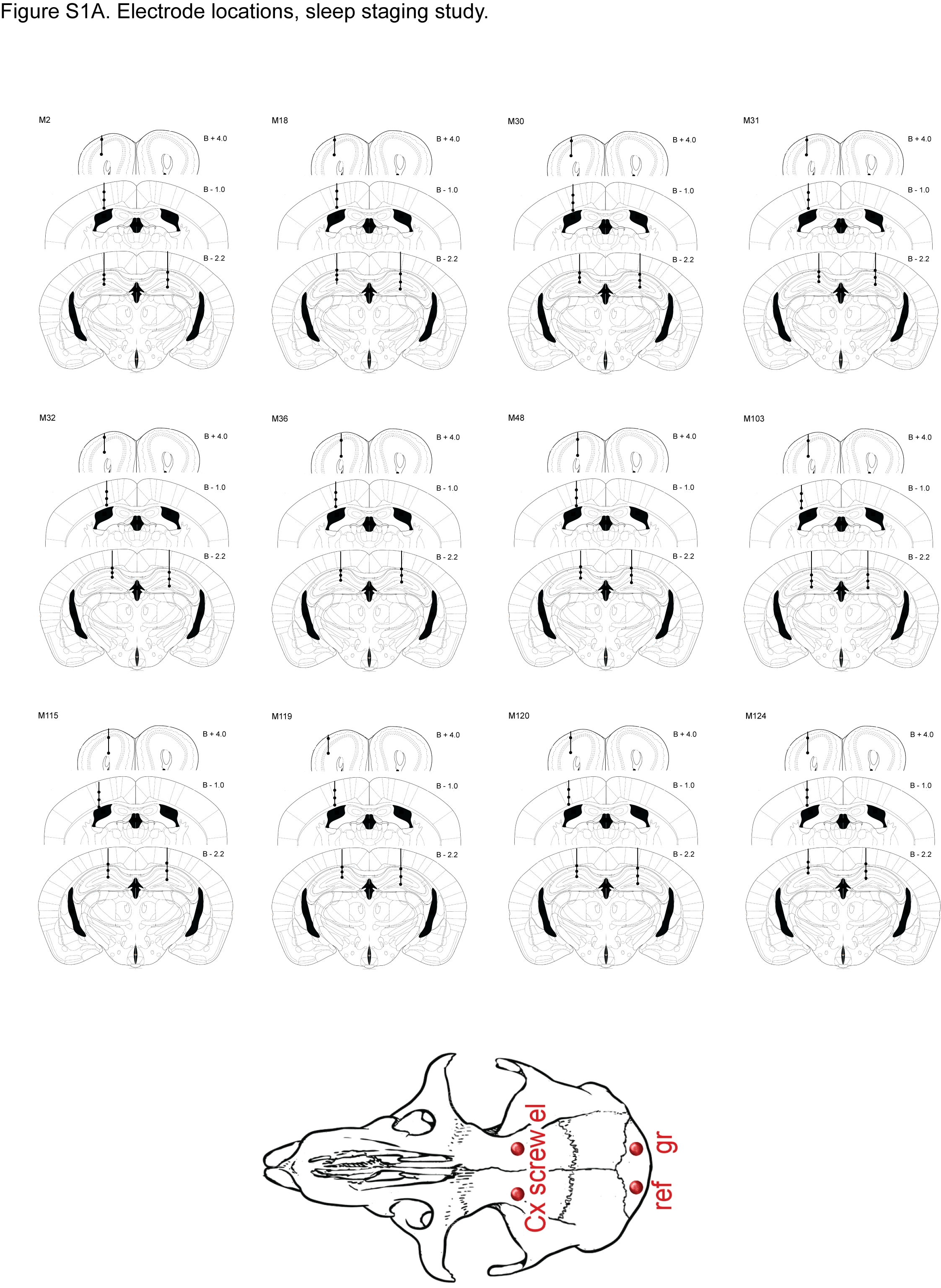

Supplement: Supplementary file 1 — Figure S1: Location of electrode tips based on electrolytic lesions and electrophysiological landmarks. (A) Mice in the main sleep staging study, (B) mice in the DSP‐4 and medetomidine study. The intracranial wire electrodes are shown individually in each mouse while the common setup of two recording bone screw electrodes and the ground and reference are shown on the bottom. [file JSR-35-e70262-s001.zip › jsr70262-sup-0001-FigureS1@Fig. S1A Electrode locations.tif]

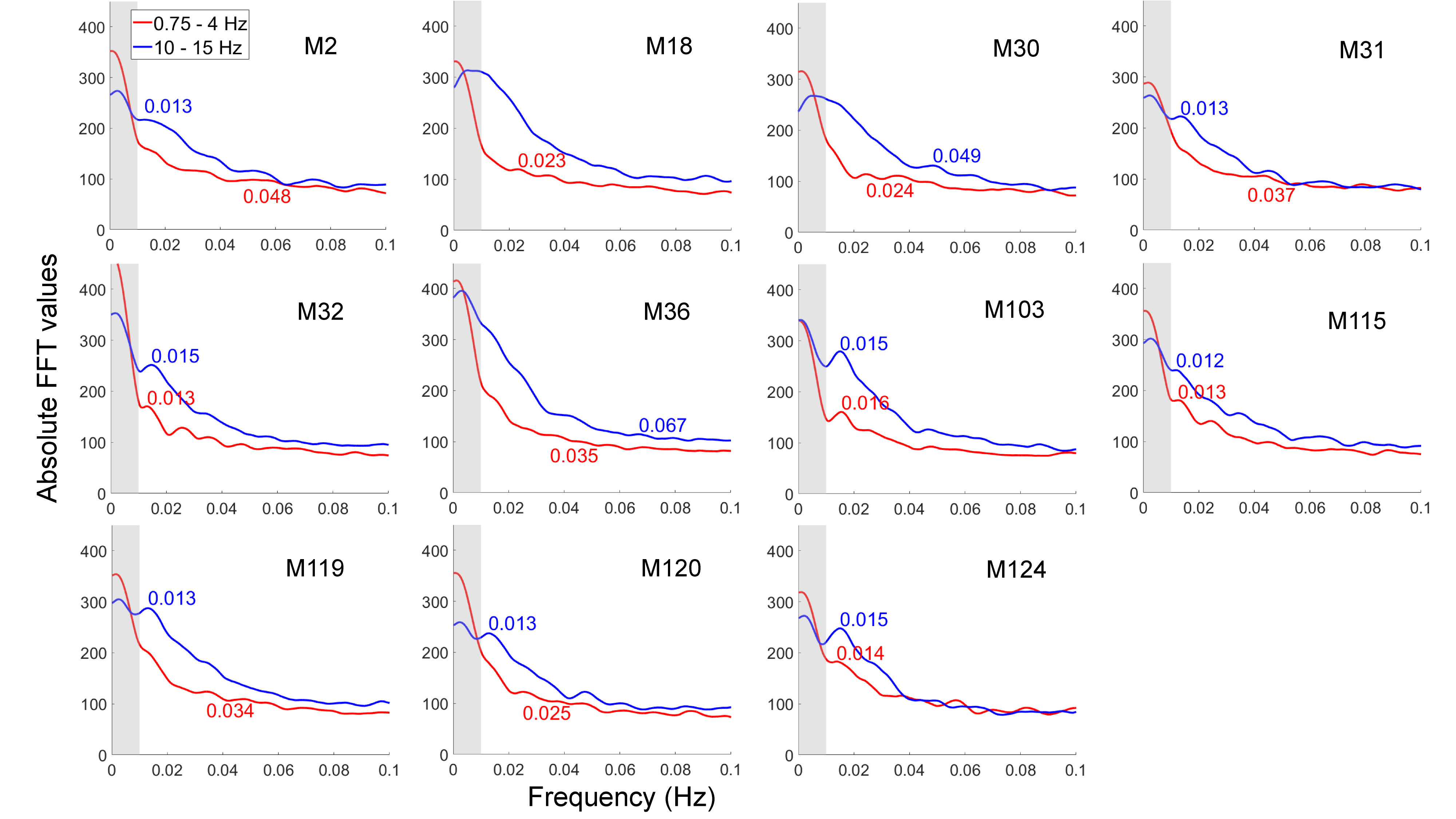

Supplement: Supplementary file 2 — Figure S2: The Identified peaks of the infra‐slow oscillation (or power fluctuation) in individual mice calculated as shown in Figure 1. The blue line is based on power fluctuations in the sigma band (10–15 Hz) while the red line shows the fluctuation in the delta or slow‐wave (0.75–4 Hz). Significant peaks are shown with numbers. The shaded area indicates that the analysis was done for putative ISO frequences between 0.01 and 0.1 Hz. [file JSR-35-e70262-s002.tif]

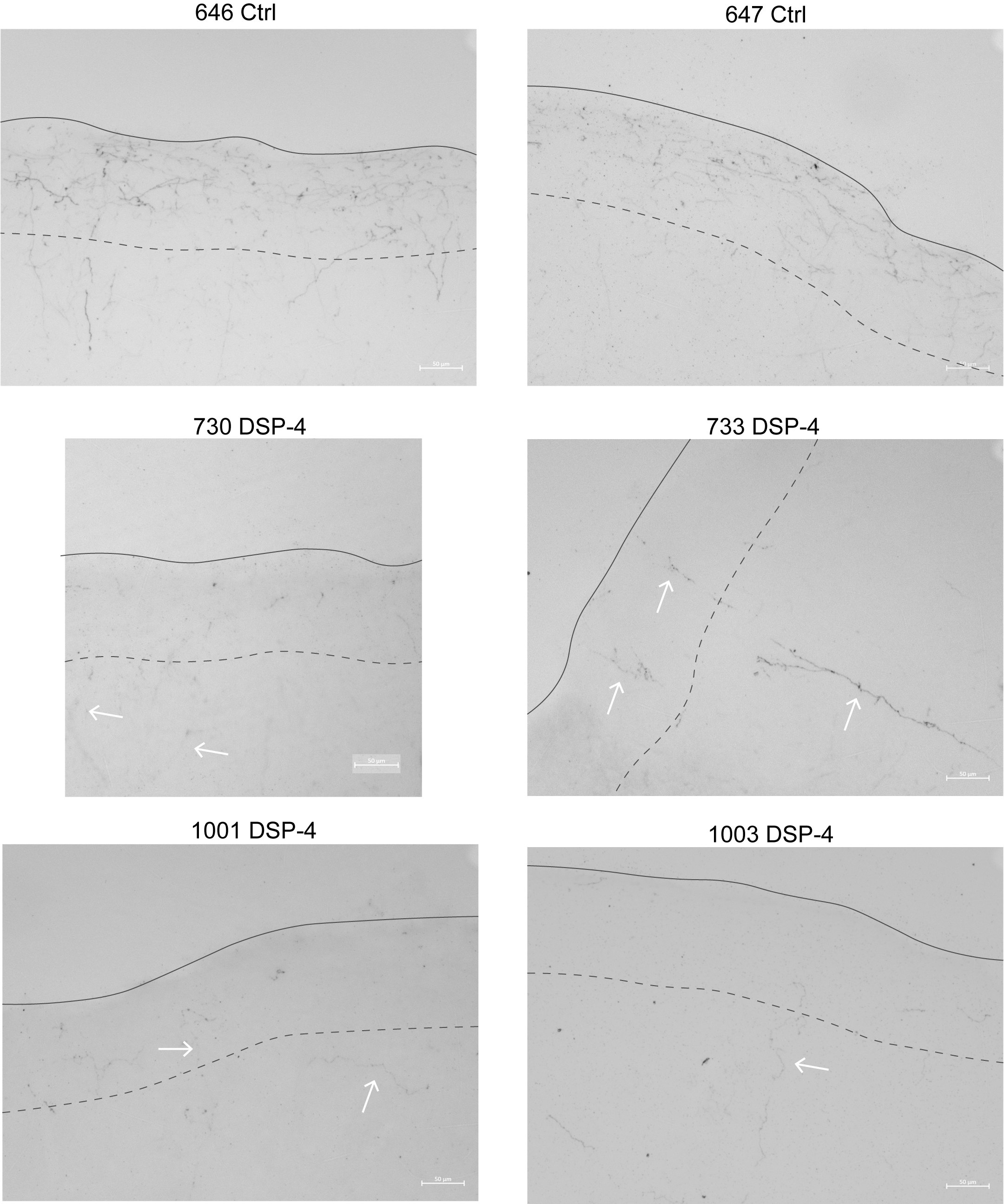

Supplement: Supplementary file 3 — Figure S3: Tyrosine hydroxylase staining with Ni‐DAB enhancement of the visual cortex in two control mice and four DSP‐4 (50 mg/kg i.p.) treated mice. The continuous line indicates the brain surface while the dashed line denotes the L1—L2 border. Note the dense meshwork of NA fibres that run mainly tangentially to the brain surface in layer 1 in the control mice. These have almost completely disappeared in the DSP‐4 treated mice. Instead, one can see individual fibres perpendicular to the brain surface (white arrows). These are probably regrowing axons of locus coeruleus NA neurons. Scale bar = 50 μm. [file JSR-35-e70262-s003.tif]
